# Overcoming Polymyxin Resistance in *Klebsiella pneumoniae* with *Ocotea* Essential Oils: Insights from In Vitro and In Vivo Analyses

**DOI:** 10.1021/acsomega.5c07706

**Published:** 2025-11-26

**Authors:** Izadora D. Faccin, Julia P. Arantes, Mariana C. Sturaro, Eduardo J. Coutinho, Danielle C. da Cruz do Nascimento, Claudia A. L. Cardoso, Flavio M. Alves, Shaline S. L. Fernandes, Nathalia da S. Damacena, Gleyce H. de Almeida de Souza, Ruana C. C. da Silva, Luana Rossato, Euclésio Simionatto, Simone Simionatto

**Affiliations:** † Laboratory of Research in Health Sciences, 186079 Federal University of Grande Dourados (UFGD), Rodovia Dourados–Itahum, Km 12, University City, ZIP Code 79804-970 Dourados, MS, Brazil; ‡ Postgraduate Program in Natural Resources (PGRN), 67708 State University of Mato Grosso do Sul (UEMS), Dourados/Naviraí, Mato Grosso do Sul, Dourados, 79950-000, Brazil; § Laboratory of Botany, Institute of Biosciences (INBIO), 54534 Federal University of Mato Grosso do Su (UFMS)l, Campo Grande, Mato Grosso do Sul 79070-900, Brazil

## Abstract

Antimicrobial resistance, particularly in carbapenem-polymyxin-resistant *Klebsiella pneumoniae* (CPR-*Kp*), is a major challenge associated with severe infections. In this study, we assessed the chemical composition and antimicrobial properties of essential oils (EOs) from *Ocotea diospyrifolia* (OdEO) and *Ocotea velloziana* (OvEO) against CPR-*Kp*. The EOs were extracted from the leaves via hydrodistillation and analyzed using gas chromatography–mass spectrometry. The antimicrobial activities of the EOs, alone and along with polymyxin B (OdEO-PMB and OvEO-PMB), were assessed through checkerboard assays, survival curves, and biofilm inhibition. Cell membrane permeability, reactive oxygen species levels, and scanning electron microscopy (SEM) were used to investigate antimicrobial mechanisms. Safety was evaluated by conducting hemolysis and toxicity tests in *Caenorhabditis elegans*. An in vivo infection model was constructed using *C. elegans* larvae to assess survival rates. OdEO and OvEO exhibited distinct chemical compositions, with α-bisabolol and viridiflorene as the major components, respectively. In silico analyses revealed that OdEO components can modulate the porin OmpK36, suggesting their ability to serve as antibiotic adjuvants. OdEO-PMB and OvEO-PMB reduced the PMB concentration required to inhibit CPR-*Kp* growth by 32-fold. Both combinations inhibited biofilm formation and caused bacterial death. The OdEO-PMB combination induced oxidative stress and increased protein leakage. Both treatments were nontoxic and nonhemolytic in the assay performed. In the infection models, OdEO-PMB and OvEO-PMB improved *C. elegans* survival rates to 72.4% and 50.9%, respectively. These results indicated that the Ocotea essential oils investigated are effective adjuvants for PMB, suggesting that they can be used to develop novel therapeutic strategies against CPR-*Kp* strains.

## Graphical abstract

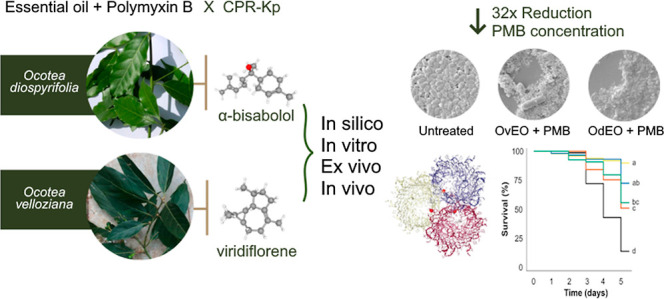

## Introduction

Antimicrobial resistance (AMR) is a critical global health concern, contributing significantly to morbidity and mortality worldwide.^1^ Given this growing threat, the World Health Organization has classified carbapenem-resistant *Klebsiella pneumoniae* as a priority pathogen because it can develop resistance to multiple classes of antibiotics, thereby challenging and limiting clinical management.^2^

Older classes of antibiotics, such as polymyxins, have re-emerged as one of the few remaining therapeutic options for carbapenem-resistant *K. pneumoniae*, although their clinical application is constrained by nephrotoxicity and neurotoxicity.^3–5^ Additionally, the increasing prevalence of polymyxin resistance mechanisms, including remodeling of the bacterial cell membrane via chromosomal alterations, such as mutations in the *mgrB* gene, and the spread of plasmid-encoded *mcr* genes, which facilitate horizontal gene transfer between bacteria, poses a significant challenge by limiting the availability of effective treatments.^6–8^

Given these limitations, innovative strategies need to be developed to combat AMR. One promising approach involves the application of conventional antibiotics with phytochemicals, such as essential oils (EOs), which has demonstrated a good tolerability profile, a wide range of biological applications, and an ability to synergistically interact with other compounds, including antibiotics.^9,10^ EOs are derived from plants and contain diverse bioactive components. They exhibit antimicrobial activity through multiple mechanisms, including disruption of the cell membrane, inhibition of protein synthesis, and interference with DNA replication.^11,12^

The genus *Ocotea* (family Lauraceae) is widely distributed across South America and is well-known for its high abundance of bioactive compounds, particularly EOs.^13^ Studies have found that species within this genus can perform various biological activities, including antimicrobial, antioxidant, antifungal, and anti-inflammatory activities.^14–16^ In this study, we investigated the effects of combining the EOs of *Ocotea diospyrifolia* and *Ocotea velloziana*, both native to Brazil, with polymyxin B against carbapenem-polymyxin-resistant *K. pneumoniae* (CPR-*Kp*).

## Results

### Gas chromatography–mass Spectrometry (GC–MS)

The EO yields (% v/w) for OdEO and OvEO were 0.25% and 0.10%, respectively. In OdEO, oxygenated sesquiterpenes were the predominant chemical group, constituting 56.1% of the total oil composition, followed by sesquiterpene hydrocarbons (31.3%) and monoterpene hydrocarbons (3.1%). In contrast, OvEO consisted of sesquiterpene hydrocarbons (68.4%) and oxygenated sesquiterpenes (13.7%). The major compounds identified in OdEO were α-bisabolol (49.7 ± 0.4%) and β-bisabolene (10.4 ± 0.4%), which together accounted for more than 50% of its composition. OvEO was characterized primarily by the presence of viridiflorene (20.1 ± 0.5%), aromadendrene (10.2 ± 0.4%), and δ-elemene (9.5 ± 0.3%). The chemical compositions of both EOs are summarized in Table 1, with the identified compounds representing 90.4% of OdEO and 82.1% of OvEO. Chromatographic profiles and mass spectra of the main constituents of OvEO and OdEO are available in the Supporting Information (Figures S1 and S2).

**Table 1:** Chemical Composition (%) of OdEO and OvEO

compounds^a^	LTPRI^b^	LTPRI^c^	concentration (%)^d^	
			OdEO	OvEO
allo-aromadendrene	1459	1460	-	2.4 ± 0.1
aromadendrene	1437	1441	-	10.2 ± 0.4
α-bisabolol	1686	1685	49.7 ± 0.4	-
α-bisabolol oxide B	1654	1658	1.7 ± 0.2	-
α-bulnesene	1507	1510	-	1.5 ± 0.2
α-cadinol	1653	1654	-	2.7 ± 0.2
α-copaene	1374	1376	-	1.6 ± 0.1
α-muurolene	1494	1500	-	1.5 ± 0.0
α-pinene	946	954	1.1 ± 0.0	-
β-bisabolene	1508	1505	10.4 ± 0.4	-
β-copaene	1433	1432	2.0 ± 0.0	-
β-elemene	1391	1390	-	5.6 ± 0.1
β-pinene	974	979	2.0 ± 0.94	-
β-selinene	1484	1490	-	1.5 ± 0.1
δ-cadinene	1520	1523	-	3.2 ± 0.2
δ-elemene	1336	1338	-	9.5 ± 0.3
epi-α-cadinol	1641	1640	-	1.2 ± 0.2
(*E*)-caryophyllene	1420	1419	2.9 ± 0.1	-
(*E*)-nerolidol	1563	1563	-	1.3 ± 0.2
γ-cadinene	1510	1513	-	3.2 ± 0.2
γ-elemene	1435	1436	7.6 ± 0.5	-
γ-muurolene	1475	1479	-	4.1 ± 0.4
γ-patchoulene	1533	1502	-	2.9 ± 0.2
globulol	1582	1590	-	6.7 ± 0.3
ledol	1631	1602	-	1.8 ± 0.2
spathulenol	1576	1578	3.1 ± 0.1	-
viridiflorol	1590	1592	1.3 ± 0.0	-
viridiflorene	1494	1496	-	20.1 ± 0.5
(Z)-β-farnesene	1456	1442	5.2 ± 0.4	-
(Z)-caryophyllene	1407	1408	3.1 ± 0.1	-
hydrocarbons monoterpenes			3.1	-
oxigenated monoterpenes			-	-
sesquiterpene hydrocarbons			31.3	68.4
oxygenated sesquiterpenes			56.1	13.7
total			90.4	82.1

a Elution order is given in DB-5 column.

b LTPRI indicates the linear-temperature-programmed retention indices that were calculated against C8–C40.

c LTPRI indicates the linear-temperature-programmed retention indices reported in the literature.

d Relative content expressed as percentage of the total composition of the oil obtained by GC–MS.

### In Silico Binding Analysis with OmpK36

The molecular screening conducted on the OmpK36 porin of *K. pneumoniae* revealed energetically favorable interactions with the major constituents of OdEO, which include α-bisabolol and β-bisabolene (Table 1). The compound α-bisabolol exhibited a binding free energy of −6.746 kcal/mol, with negative contributions from van der Waals (−13.580) and electrostatic (−6.964) interactions, indicating conformational stability within the formed complex. Additionally, α-bisabolol deeply accommodates the channel cavity, a critical region for permeability, stabilized by negative van der Waals and electrostatic interactions, resulting in a thermodynamically favorable complex (Δ*G* = −6.746 kcal/mol) (Figure 1A).

**Figure 1:**
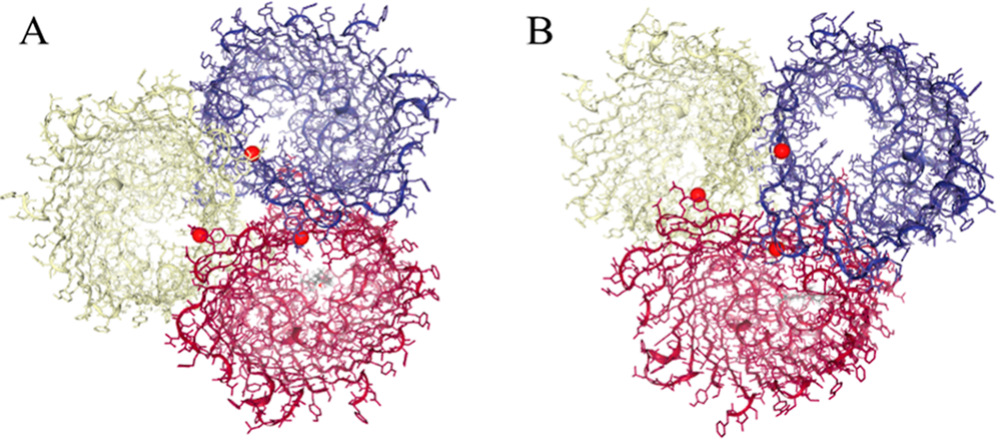
Three-dimensional structure of the OmpK36 porin from *K. pneumoniae* shows the predicted binding sites (in red) of the major constituents of *Ocotea* essential oil. (A) α-Bisabolol is positioned within the lumen of the β-barrel channel. (B) β-Bisabolene is also present in this region. The β-barrel represents the central transmembrane domain of the porin, forming a channel through which molecules can pass. In both cases, the ligands interact with residues lining the β-barrel. Each color represents an individual polypeptide chain: purple for one subunit, red/white for the second identical subunit, and yellow for the third identical subunit.

In contrast, β-bisabolene has a more negative binding free energy (−7.355 kcal/mol), characterized by a strong hydrophobic component (van der Waals energy = −15.677) and a lower contribution of electrostatic interactions (−1.067), reflecting predominantly nonpolar interactions with the protein surface. β-Bisabolene occupies an overlapping binding site, anchoring also at the porin channel, as a result of its hydrophobic interactions, as indicated by the dominant van der Waals component of the binding energy (Δ*G* = −7.355 kcal/mol) (Figure 1B).

### Antimicrobial Activity

The broth microdilution assay determined MICs ≥256 μg/mL for OdEO and OvEO, whereas PMB exhibited an MIC of 64 μg/mL against CPR-*Kp*. Individually, OdEO and OvEO were ineffective, with no antimicrobial activity, as was PMB at concentrations below 64 μg/mL. However, when combined, OdEO-PMB and OvEO-PMB exhibited strong additive and synergistic effects, with FICI values of 0.53 and 0.28, respectively (Table 2). These findings were further supported by SynergyFinder analysis, which revealed inhibition rates exceeding 80% for the combinations, along with a ZIP score <10 for OdEO-PMB (additive interaction) and a ZIP score >10 for OvEO-PMB (synergistic interaction) (Figure 2).

**Table 2:** Antimicrobial and Checkerboard Results of the Combination of OdEO and OvEO with PMB Against CPR-*Kp* ^a^

	MIC (μg/mL)					
	alone	associated				
*Ocotea*	EO	PMB	EO	PMB	FICI	interaction
**OdEO**	>256	64	256	2	0.53	additive
**OvEO**	>256	64	128	2	0.28	synergic

a Subtitle: EO = essential oil; PMB = polymyxin B; MIC = minimum inhibitory concentration; FICI = fractional inhibitory concentration.

**Figure 2:**
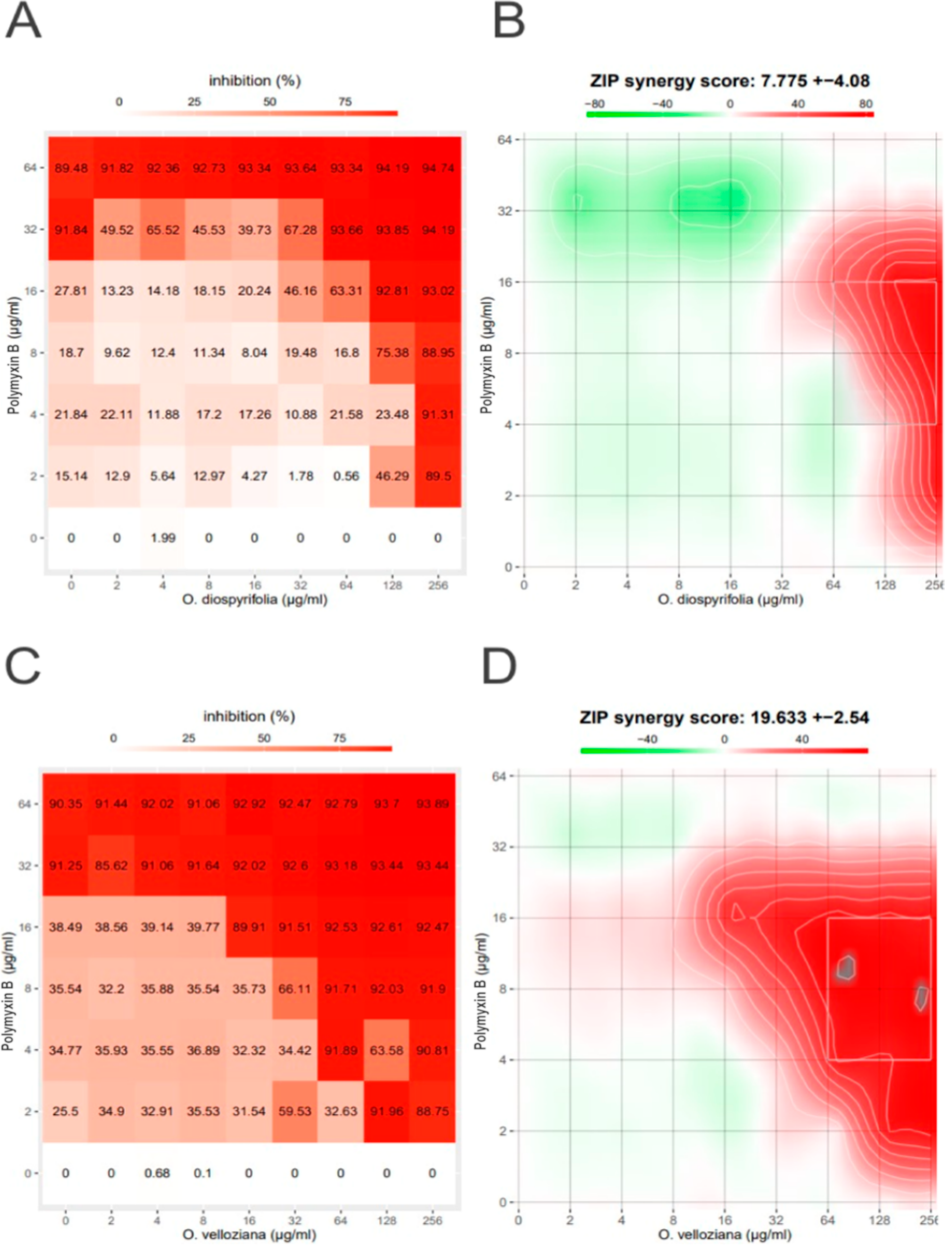
Synergy finder analysis. (A) Dose–response matrix for OdEO-PMB. (B) ZIP synergy score (7.775 ± 4.08) for OdEO-PMB. (C) Dose–response matrix for OvEO-PMB. (D) ZIP synergy score (19.633 ± 2.54) for OvEO-PMB. The red areas indicate better dose combinations related to bacterial growth inhibition (inhibition >80%).

Although the EOs individually showed no antimicrobial activity against the planktonic cells of CPR-*Kp* at the tested concentrations, their inhibitory effect on biofilm formation was statistically comparable to that of the negative control (no biofilm formation), the combination groups, and PMB at 64 μg/mL (*p* > 0.05). PMB at 2 μg/mL had a less pronounced antibiofilm effect than the positive control, although its activity was not comparable to that of the combination groups (Figure 3).

**Figure 3:**
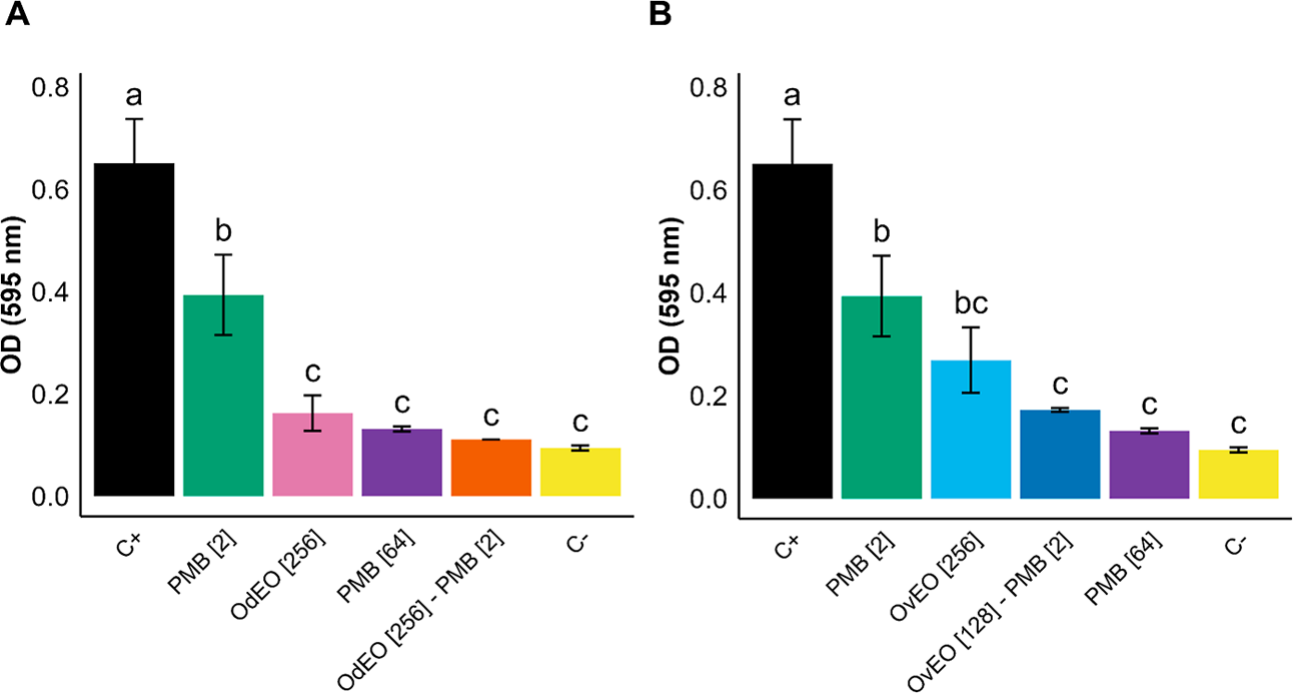
Antibiofilm activity against CPR*-Kp*. (A) Effects of OdEO and PMB, alone and in combination. (B) Effects of OvEO and PMB, alone and in combination. The concentrations (μg/mL) are indicated in brackets. Different letters indicate statistically significant differences (*p* < 0.05); identical letters indicate no significant difference.

In the survival curve assay, no statistically significant differences were found between OdEO-PMB, OvEO-PMB, and PMB at 64 μg/mL (*p* > 0.05). However, both OdEO-PMB and OvEO-PMB showed statistically significant differences compared to PMB at 2 μg/mL and the EOs alone over a 24 h period (*p* < 0.05), which indicated that a synergistic interaction occurred. Unlike the combinations, 2 μg/mL PMB suppressed bacterial growth for only 10 h (Figure 4A). The results from the spotting assay revealed that OdEO and OvEO, when used individually, did not exhibit antimicrobial activity, whereas their combination with PMB synergistically reduced visual colony formation within the 1:50 dilution growth (Figure 4B).

**Figure 4:**
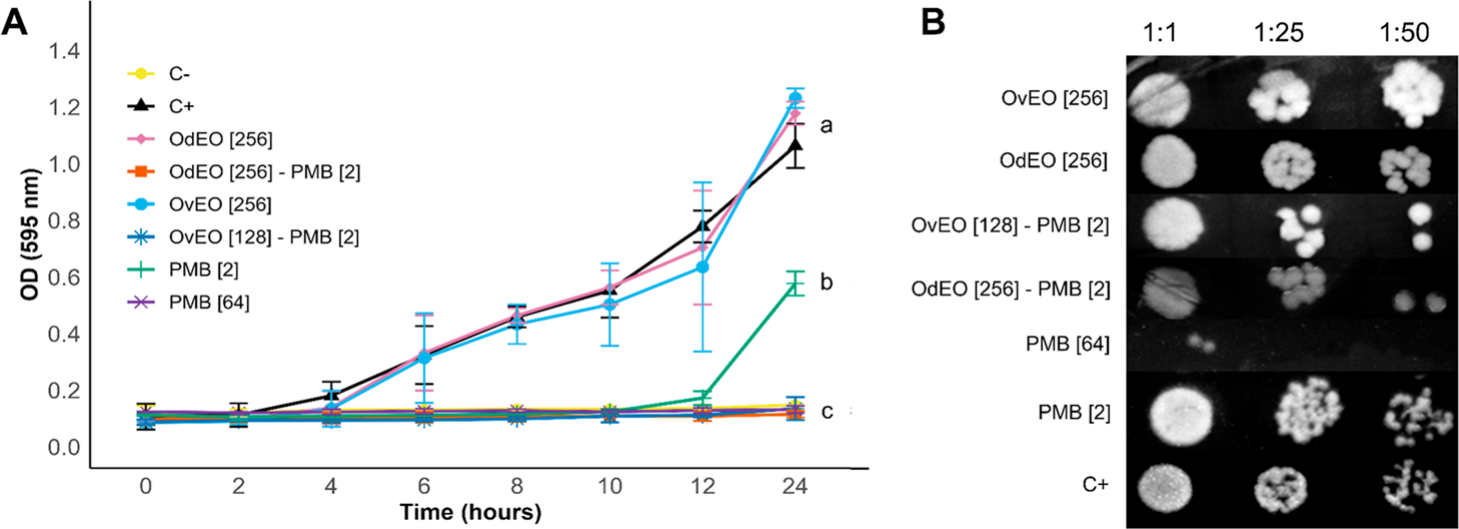
Antimicrobial activity of OdEO-PMB, OvEO-PMB, and compounds in isolation against CPR-*Kp* was evaluated. (A) Survival curve; different letters indicate statistically significant differences (*p* < 0.05). (B) Spotting test; the concentrations (μg/mL) are indicated in brackets.

### Cell Membrane Permeability Assay

The cell membrane permeability assay revealed no detectable protein extravasation in CPR-*Kp* treated with PMB at 2 μg/mL, OvEO-PMB, or the isolated EOs (Figure 5A). In contrast, OdEO-PMB triggered detectable protein release only after 4 h of exposure, indicating that initial interactions induce limited membrane perturbations, while prolonged exposure leads to cumulative destabilization and the formation of lesions large enough to allow macromolecule leakage. Although PMB at 64 μg/mL induced measurable protein release, a decrease in protein concentration was found after 4 h of incubation. This decrease occurred probably due to protein instability in the medium, leading to degradation over time and subsequently affecting quantification. No significant differences were found among all treatment groups after 4 h (*p* > 0.5).

**Figure 5:**
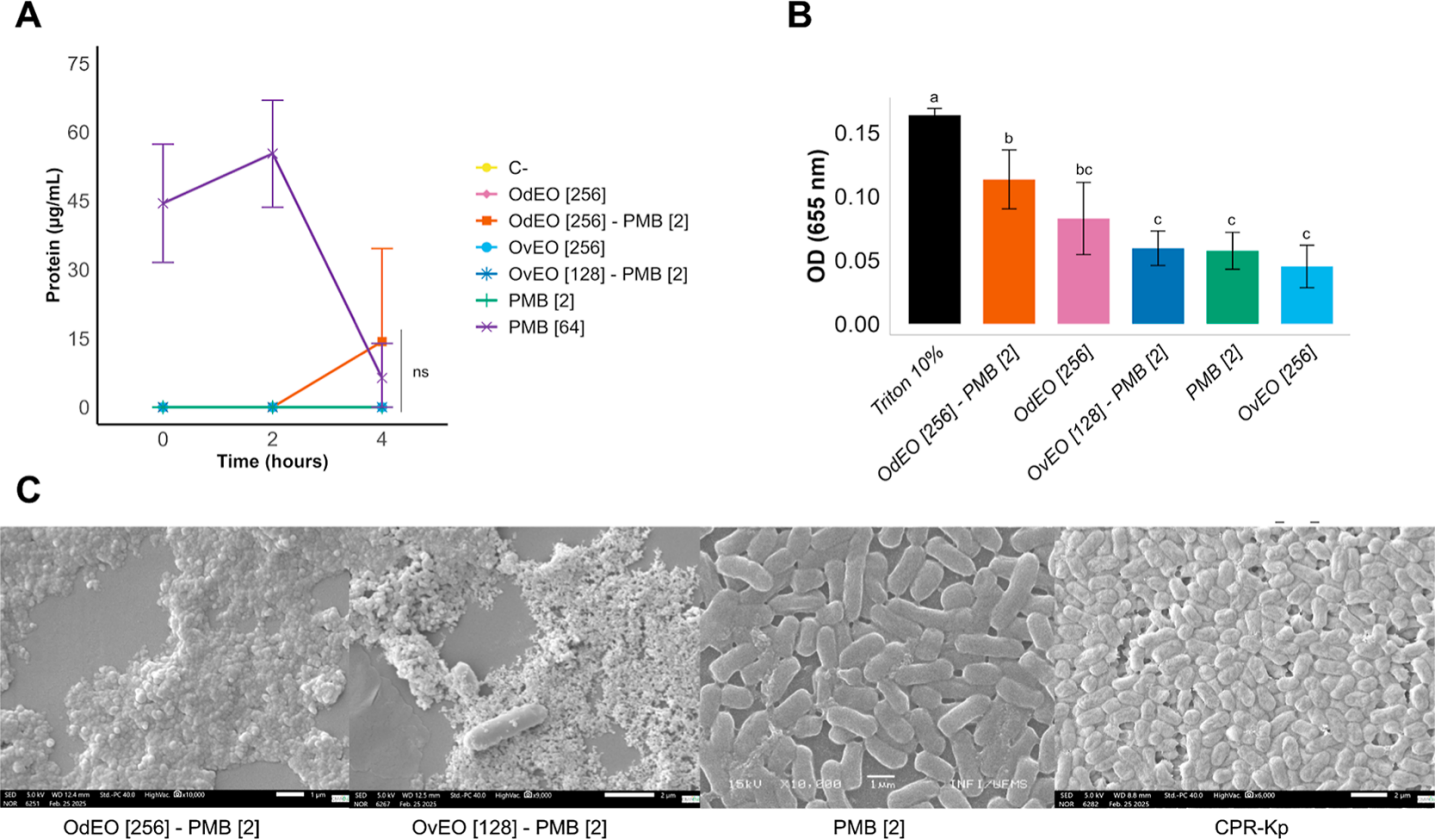
Effects of combinations on CPR-*Kp* were evaluated by the (A) quantification of protein extravasation, (B) quantification of ROS, and (C) SEM images of CPR-*Kp* and treatments. The concentrations (μg/mL) are indicated in brackets. Different letters indicate significant differences (*p* < 0.05); identical letters (or ns) indicate no significant difference.

### Quantification of ROS

We quantified ROS to assess the oxidative potential of OdEO-PMB and OvEO-PMB in CPR-*Kp* cells (Figure 5B). While none of the treatments resulted in significant differences compared to the positive control, the OdEO-PMB combination resulted in a substantial increase in ROS production, which was statistically similar to that of the isolated EO. This observation suggests that PMB effectively enhances the oxidative potential of OdEO, inducing significant stress in CPR-*Kp* cells and contributing to its antimicrobial effects. In contrast, 2 μg/mL PMB resulted in ROS levels comparable to those of both the OvEO-PMB combination and the isolated EOs, indicating a more moderate oxidative response.

### SEM Analysis

We performed SEM analysis to assess the effects of the treatments on the bacterial cells (Figure 5C). Images of CPR-*Kp* cells exposed to OdEO-PMB and OvEO-PMB revealed inhibited bacterial growth and clear signs of cell death, characterized by the presence of cellular debris. In contrast, treatment with 2 μg/mL PMB did not inhibit growth or cause structural damage, similar to the positive control, in which CPR-*Kp* was cultured in BHI medium.

### Hemolysis Assay

To evaluate ex vivo cellular toxicity, a hemolysis test was performed to determine whether the compounds tested could destabilize the erythrocyte membrane, resulting in cell lysis. The hemolysis rates were 8.30% for OdEO and 2.83% for OvEO (Figure 6). However, OdEO-PMB and OvEO-PMB exhibited negligible hemolytic activity due to their lower doses, with rates that were not significantly different from those of the PMB and D-PBS controls (*p* > 0.05).

**Figure 6:**
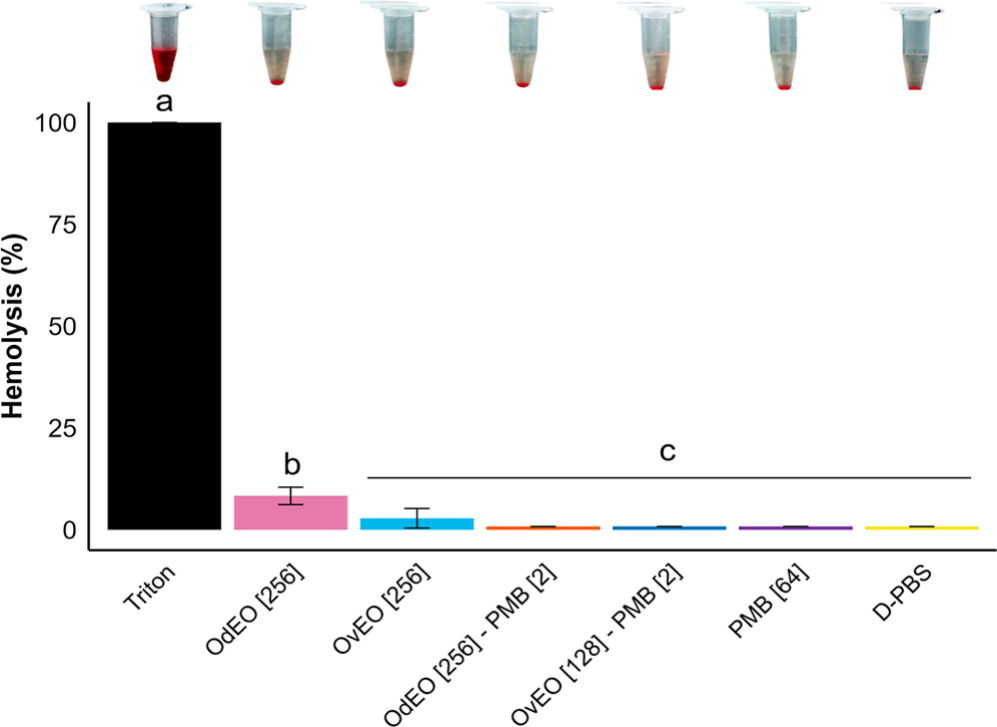
Hemolysis rates of OdEO-PMB, OvEO-PMB, and the isolated compounds are shown. The concentrations (μg/mL) are indicated in brackets. Different letters indicate statistically significant differences (*p* < 0.05); identical letters indicate no significant difference.

### In vivo Toxicity Assay

The safety assay performed on the wild-type strain *Caenorhabditis elegans* (N2) revealed no significant differences (*p* > 0.05) in survival rates among the evaluated treatments and the control group (Figure 7A). The OdEO-PMB and OvEO-PMB combinations yielded high survival averages of 97.91% and 95.44%, respectively. PMB at 64 μg/mL and TGC at 64 μg/mL resulted in less pronounced survival rates of 90% and 90.7%, respectively. These findings suggested that the tested combinations were well-tolerated and did not significantly compromise organismal survival, highlighting their potential safety for therapeutic applications.

**Figure 7:**
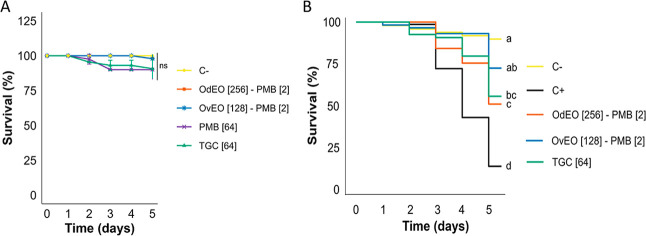
Kaplan–Meier survival curves of *C. elegans* models are presented. (A) Toxicity was assessed using the N2 strain. PMB and TGC were included as antibiotic controls, whereas nematodes treated with only M9 buffer served as the negative control (C−). (B) Infection control was assessed using the AU37 strain infected with CPR-*Kp*. The treatments included OdEO-PMB and OvEO-PMB. Infected but untreated nematodes served as the positive control (C+), TGC served as a standard antibiotic, and uninfected nematodes treated with M9 buffer served as the negative control (C−). Different letters indicate statistically significant differences (*p* < 0.05); identical letters indicate no significant (ns) difference.

### In vivo Infection Model

The in vivo assay conducted using nematodes infected with and treated with the OdEO-PMB combination showed an average survival rate of 72.4%. This result was not significantly different from that of the uninfected control group (90%) or the group treated with the reference antibiotic TGC (55.6%). In contrast, treatment with the OvEO-PMB combination yielded a survival rate of 50.9%, which was statistically similar to that of the TGC group but significantly lower than that of the OdEO-PMB group. Both OdEO-PMB and OvEO-PMB resulted in significantly higher survival rates compared to the infected untreated control group (13.6%; *P* < 0.05), highlighting the therapeutic potential of these EO combinations in managing nematode infections (Figure 7B).

## Discussion

Given that the threat posed by AMR is increasing, the development of alternative antimicrobial therapeutic techniques has become crucial. In 2019, nearly five million deaths were associated with resistant infections, with *K. pneumoniae* identified as one of the six leading pathogens, making AMR a major global cause of mortality.^17^ Although this crisis has worsened, the discovery of new antimicrobial agents has slowed considerably over the past few decades. Most novel antibiotics introduced in recent years are modifications of existing classes, with few truly innovative agents reaching the market.^18^ Therefore, many researchers consider the application of natural compounds, such as plant EOs, and their combination with antibiotics as a promising approach to combat multidrug-resistant bacteria.^19^

To address this need, we investigated the synergistic interplay between OdEO-PMB and OvEO-PMB against a CPR-Kp strain harboring alterations in the mgrB gene, an important determinant of polymyxin resistance. Given the limited number of studies addressing this topic, deciphering the synergistic potential of OdEO-PMB and OvEO-PMB may offer valuable insights into the development of alternative therapeutic strategies against CPR-*Kp* pathogens.

We found that OdEO is characterized by high levels of sesquiterpene hydrocarbons and oxygenated sesquiterpenes, with α-bisabolol being the predominant compound. This sesquiterpene has various biological effects, including anticancer, anti-inflammatory, antimicrobial, and antioxidant effects.^20^ Additionally, the sesquiterpene β-bisabolene, which is present in significant amounts, is a potential antitoxic and antitumor agent. It may also act synergistically with ampicillin against *Staphylococcus aureus*.^21,22^ OvEO is predominantly composed of viridiflorene, aromadendrene, and δ-elemene. These compounds are known constituents of EOs and may have antimicrobial and antifungal properties.^23,24^

Using a structural biology perspective, we performed docking simulations suggesting that α-bisabolol and β-bisabolene, the main components of OdEO, may interact with regions of the OmpK36 porin of *K. pneumoniae*. While these profiles resemble findings reported for *E. coli* OmpF.^25^ they remain predictive and lack experimental validation. As porin alteration is a common resistance mechanism in Gram-negative bacteria, the hypothesis that natural compounds act as reversible, nongenetic modulators is important^26^ requiring further molecular and functional studies.

In this study, OdEO-PMB and OvEO-PMB exhibited additive and synergistic interactions, respectively, as indicated by the FICI and ZIP score. Both combinations led to a 32-fold reduction in the PMB MIC. Considering the concerns regarding toxicity and increasing resistance to PMB, these findings suggest that OdEO-PMB and OvEO-PMB can help mitigate such limitations by significantly lowering the dose of PMB, thereby reducing the risk of adverse effects while enhancing antibacterial efficacy.^27^

The tested combinations also exhibited CPR-*Kp* antibiofilm activity, addressing a particularly concerning form of bacterial resistance.^28^ This property makes them valuable for treating biofilm-associated infections, such as chronic rhinosinusitis and chronic wounds, which are extremely difficult to manage and often require prolonged treatment that can significantly affect the health of patients.^29^ Therefore, discovering new candidates to combat bacterial biofilms, such as OdEO-PMB and OvEO-PMB, is necessary for advancing treatment strategies and improving patient outcomes.

EOs are interesting antimicrobial agents as they target multiple bacterial processes simultaneously. This approach not only improves bacterial killing but also reduces the chances of developing resistance, preserving the effectiveness of last-resort antibiotics such as polymyxins.^30^ OdEO-PMB induced cellular damage in CPR-*Kp* with detectable protein release after 4 h of exposure, and also triggered oxidative stress. The hydrophobic nature of essential oils facilitates their insertion into the lipid bilayer, leading to disorganization of membrane components and loss of integrity, while ROS generation may further compromise cell wall through lipid peroxidation and protein oxidation, thereby amplifying membrane destabilization and accelerating cell death.^31^ This destabilization is a time- and dose-dependent process, as different constituents require specific conditions to induce structural impairment, which increases progressively with exposure duration and concentration.^32,33^ The reduced effect observed for OvEO-PMB may be attributable to the lower concentration tested and to differences in its chemical composition, suggesting that the antimicrobial activity of *Ocotea* essential oils is strongly influenced by both dose and phytochemical profile.

To evaluate the safety parameters of the combinations, hemolysis assays and in vivo toxicity assessments were performed. The isolated *Ocotea* EOs showed low hemolytic activity, while both OdEO-PMB and OvEO-PMB presented negligible hemolysis, confirming their hemocompatibility within the accepted 5% threshold.^34^ Consistently, no reduction in nematode lifespan was observed, reinforcing that the combinations are well tolerated at the organism level. *C. elegans* was employed as it represents a cost-effective and ethically accepted in vivo model with conserved stress and detoxification pathways, widely used for preliminary toxicity screening of antimicrobial compounds.^35^ The agreement between ex vivo and in vivo assays strengthens the evidence of biocompatibility, although complementary evaluation in mammalian systems remains necessary for translational relevance.

In vivo experimentation is crucial for testing new antimicrobial strategies, as it confirms their efficacy in a complex living system, bridging in vitro findings to real-world application.^36^ In this study, OdEO-PMB and OvEO-PMB maintained their synergistic profile in the *C. elegans* infection model, significantly increasing the survival rates of nematodes and overcoming CPR-*Kp* infection. These findings reinforce that *Ocotea* EOs are effective adjuvants, supporting their further development for combating resistant bacterial infections.

The results of this study agree with the findings reported by Pimentel et al., who also identified α-bisabolol (45.8%) and β-bisabolene (9.4%) as the major constituents of OdEOs.^14^ Their study revealed the synergistic activity of this EO in combination with ampicillin against CPR-*Kp*. The mechanism of action involved protein leakage after 2 h of exposure, with a significant survival rate of 85% in a *C. elegans* infection model. In contrast, research on the antimicrobial properties of OvEO is limited. Its primary documented activity is as a larvicidal agent against *Aedes aegypti*.^25^ EOs from other species within the genus *Ocotea* have demonstrated promising antibacterial and antifungal effects, indicating that *O. velloziana* may also be promising in this regard.^37,38^

The use of EOs as therapeutic alternatives is limited by the variability in their chemical composition, which is influenced by factors such as the harvest period and environmental conditions, along with their volatility and instability.^39^ Chemical synthesis and the use of nanotechnology are promising strategies to overcome these limitations and harness the therapeutic potential of these oils.^40^

This study demonstrated that OdEO and OvEO are promising adjuvants to combat CPR-*Kp*. Additive and synergistic interactions between OdEO-PMB and OvEO-PMB reduced the PMB MIC by 32-fold, addressing toxicity concerns while enhancing antimicrobial efficacy. Additionally, the combinations revealed antibiofilm activity. Safety assessments from ex vivo and in vivo assays confirmed the nontoxic nature of these combinations. However, the findings on the mechanisms of action, as well as the in vivo assays in *C. elegans*, should be regarded as preliminary. Future studies should broaden toxicity and antimicrobial evaluations in additional in vivo models, while also focusing on formulation optimization and advanced in vivo investigations to translate these results into potential clinical applications.

## Material and Methods

### Plant Material

Leaves of *O. diospyrifolia* and *O. velloziana* were collected from a native protection park area in Naviraí, Mato Grosso do Sul, Brazil (23°03′37″ S; −54°11′13 W) in 2022. Specimens of *O. diospyrifolia* (voucher number 3762) and *O. velloziana* (voucher number 84591) were identified and deposited in the herbarium of the Federal University of Grande Dourados. The collection and associated research were registered with the National System of Genetic Resource Management and Associated Traditional Knowledge (SISGEN) under registration numbers A5BB263 and A9B7A92.

### Essential Oils Extraction

Leaves from *O. diospyrifolia* and *Ocotea vannamei* (500 g each) were subjected to a seven-type apparatus, following the guidelines provided in the European Pharmacopeia [17]. The process involved adding 4 L of water and was conducted over 240 min. The EOs obtained were dried using anhydrous sodium sulfate to remove residual moisture. Each extraction was performed at least three times to ensure that the results were consistent and reproducible. The yield of the EOs was calculated based on the mass of the fresh plant material and expressed as a percentage (% m/m). The EOs from *O. diospyrifolia* and *O. velloziana* were designated OdEO and OvEO, respectively, and were stored at 4 °C in sealed containers until further use.

### Gas chromatography–mass Spectrometry (GC–MS)

Samples of OdEO and OvEO (100 μg/mL) were analyzed. The analysis was conducted using a GC-2010 Plus gas chromatograph (Shimadzu, Kyoto, Japan) connected to a mass spectrometer (GC–MS 2010 Ultra) equipped with a DB-5 column (J and W, Folsom, California, USA). The column consisted of capillary-fused silica coated with 5% phenyl dimethylpolysiloxane, measuring 30 m in length, 0.25 mm in internal diameter, and 0.25 μm in film thickness. For the analysis, 1 μL of the sample was injected in the split mode (1:20). The heating program involved starting at 50 °C, increasing at a rate of 3 °C/min to 280 °C, and holding the initial temperature for 10 min. The injector temperature was maintained at 250 °C.

The mass spectrometry parameters were set to an electron impact ionization voltage of 70 eV, with MS scanning in the *m*/*z* range of 45–600 and a scan time of 0.3 s. Retention indices were calculated by analyzing a standard C6–C30 alkane mixture (Sigma-Aldrich; purity ≥90%) under the same conditions. The compounds were identified by comparing retention indices with values reported in published studies and interpreting the obtained mass spectra. Additionally, comparisons were made with the NIST21 and WILEY229 spectral databases.^41^ The peak areas for each compound were determined through manual integration of total ion chromatograms, and the areas were converted into relative percentage values.^42^

### Molecular Docking Studies

To determine whether the major components of OdEO (α-bisabolol and β-bisabolene) influence its activity against *K. pneumoniae*, molecular docking studies were performed. The interactions between these compounds and the OmpK36 porin were predicted using the DockThor-VS v2.0 platform, a widely used tool for structure-based virtual screening of protein–ligand interactions.^43^ The three-dimensional structure of the target protein OmpK36 was obtained from the Protein Data Bank (PDB ID: 5O79).^44^ The protein chain was automatically prepared using the DockThor platform. The ligands α-bisabolol (CID: 10586) and β-bisabolene (CID: 123034) were obtained in the SDF format from the PubChem database.^45^ Their molecular structures were converted to the PDBQT format using an automated Python script. A blind docking strategy was adopted, covering the entire protein surface to identify potential binding sites. The search grid was automatically defined by the DockThor tool to include the entire protein-binding box. For each ligand, 24 independent docking runs were performed with different spatial sampling regions and the default algorithm parameters. The resulting protein–ligand complexes were ranked based on the predicted binding free energy (in kcal/mol) and visualized using PyMOL v2.5.4.

### Bacterial Strain

The CPR-*Kp* strain used in this study was isolated and characterized in another study.^46^ Bacterial species identification and antimicrobial resistance profiling were conducted using the Phoenix Automated System (BD Diagnostic Systems, Sparks, MD, USA). To determine the molecular basis of polymyxin resistance, whole-genome sequencing was performed. For the experiments, the strain was grown on brain heart infusion (BHI) agar and incubated at 37 °C for 24 h.

### Antimicrobial Activity Test

The minimum inhibitory concentration (MIC) of OdEO and OvEO, along with the antibiotic polymyxin B (PMB), was evaluated using a microdilution method following the guidelines provided by the Clinical and Laboratory Standards Institute (CLSI).^47^ The PMB solution (catalog number 102450080, source BCCG2613) was obtained from Sigma (St. Louis, USA) and prepared following the manufacturer’s protocol. Stock solutions of OdEO and OvEO were prepared in dimethyl sulfoxide (DMSO_PA ACS) at concentrations not exceeding 0.5%. The bacterial suspensions of CPR-*Kp* were adjusted to the 0.5 McFarland standard and diluted 1:100, resulting in a concentration of 1.5 × 10^6^ colony-forming units (CFU)/mL. Positive controls (untreated bacterial cultures) and negative controls (BHI broth only, to confirm sterility) were included in the procedure. The plates were incubated at 37 °C for 24 h, and the MIC was identified as the lowest concentration of each treatment capable of inhibiting bacterial growth.

### Checkerboard Assay

To assess the synergistic effects of combining OvEO and OdEO with PMB, designated OvEO-PMB and OdEO-PMB, against CPR-*Kp*, a checkerboard assay was conducted. The PMB concentrations ranged from 0.25 μg/mL to 64 μg/mL, whereas the OdEO and OvEO concentrations varied between 0.5 μg/mL and 256 μg/mL. Serial dilutions of the EOs were prepared horizontally across the wells of a microdilution plate, while PMB serial dilutions were arranged vertically. Positive controls, consisting of untreated bacterial strains, and negative controls, consisting of only BHI broth to confirm sterility, were included. The bacterial suspensions were standardized to a 0.5 McFarland scale and diluted 1:100 (1.5 × 10^6^ CFU/mL) before inoculation. Then, the plates were incubated at 37 °C for 24 h. The fractional inhibitory concentration index (FICI) was calculated using the following equation: before inoculation. Then, the plates were incubated at 37 °C for 24 h. The fractional inhibitory concentration index (FICI) was calculated using the following equation

FICI=(FICA+FICB)

Where

$$FIC\;A=\frac{MIC\;of\;drug\;A\;in\;combination}{MIC\;of\;isolated\;drug\;A}$$

$$FIC\;B=\frac{MIC\;of\;drug\;B\;in\,\;combination}{MIC\;of\;isolated\;drug\;B}$$

The results were interpreted based on ΣFICI values, which were classified as follows: synergistic (FICI ≤0.5), additive (0.5 < FICI ≤1.0), noninteractive (1.0 < FICI ≤4.0), and antagonistic (FICI >4.0) [21]. Data from the checkerboard assay were further analyzed using the zero-interaction potency (ZIP) model via SynergyFinder software. ZIP scores were categorized as synergistic (>10), additive (−10 to 10), or antagonistic (<−10), providing a second quantitative assessment of interactions between treatment combinations.^48^

### Biofilm Formation Inhibition

The ability of OdEO and OvEO at 256 μg/mL and that of PMB at 64 μg/mL and 2 μg/mL to inhibit CPR-*Kp* biofilm formation was assessed. The effects of the combinations of OdEO-PMB (OdEO, 256 μg/mL with 2 μg/mL PMB) and OvEO-PMB (OvEO, 128 μg/mL with 2 μg/mL PMB) were also evaluated. The plates were incubated under static conditions at 37 °C for 24 h to allow bacterial biofilm formation and maturation. After incubation, planktonic cells were removed by serial washing with distilled water, and biofilms were stained with crystal violet (0.1%) for 20 min, following previously established protocols.^49^ Excess dye was washed off, and the stained biofilms were solubilized in 70% ethanol. The mass of the biofilm was quantified by measuring the absorbance at 595 nm using an absorbance reader (iMark Microplate, Bio-Rad, São Paulo, SP, Brazil). Untreated CPR-*Kp* served as the positive control, whereas BHI broth was used as a sterility control (negative control).

### Survival Curve and Spotting Assay

The changes in the survival of CPR-*Kp* were analyzed in the presence of EOs and PMB, both individually and in combination, at their respective MICs. A positive control (untreated CPR-*Kp*) and a negative control (BHI broth to confirm sterility) were included in the experiment. Bacterial survival was monitored at 0, 2, 4, 6, 8, 10, 12, and 24 h postinoculation by measuring the optical density at 595 nm using an absorbance reader (iMark Microplate, Bio-Rad, São Paulo, SP, Brazil). The antimicrobial activity of the combinations was further evaluated by conducting a spotting assay. For this, 5 μL aliquots of the tested compounds, along with the bacterial inoculum, were plated onto Mueller–Hinton agar. Sterility controls (0.9% saline and culture medium) and a positive control (culture medium with untreated bacterial suspension) were included. Viability was assessed after 24 h of incubation at 37 °C.^14^

### Cell Membrane Permeability

The effect of the combination treatment on the cell membrane permeability of CPR-*Kp* was analyzed by measuring protein leakage into the supernatant. The bacterial suspensions were exposed to the MICs of OvEO-PMB, OdEO-PMB, and the individual compounds (OvEO, OdEO and PMB). The samples were incubated at 37 °C, and aliquots were collected at 0, 1, 2, and 4 h. At each time interval, the samples were centrifuged at 2500 rpm for 5 min at 4 °C. After centrifugation, 25 μL of the supernatant was transferred to a flat-bottom 96-well plate, and 200 μL of BCA working reagent (Pierce BCA Protein Assay) was added to each well. The plate was incubated at 37 °C for 30 min, and the absorbance was measured at 595 nm. A saline solution containing bacteria served as the negative control.

### Quantification of Reactive Oxygen Species (ROS)

To evaluate the oxidative potential of OdEO-PMB and OvEO-PMB in bacterial cells, reactive oxygen species (ROS) levels were measured by conducting a nitro blue tetrazolium (NBT) assay. Briefly, 100 μL of bacterial culture was exposed to 500 μL of OdEO-PMB, OvEO-PMB, or individual compounds, which served as controls. The cultures were incubated at 37 °C for 6 h. Next, the bacterial cells were harvested via centrifugation at 10,000*g* for 10 min at 4 °C. The resulting pellet was resuspended in a 2% NBT solution and incubated for 1 h at room temperature in the dark. After incubation, the mixture was centrifuged at 8000*g* for 2 min, and the supernatant was removed. The pellet was washed twice, first with PBS and then with methanol, followed by centrifugation at 8000*g* for 2 min after each wash. To disrupt the cell membranes, the pellet was treated with 2 M KOH. A 50% DMSO solution was added, and the samples were incubated at room temperature for 10 min to dissolve the formazan crystals. The mixture was centrifuged again at 8000*g* for 2 min, and 100 μL of the supernatant was transferred to a 96-well plate. The absorbance was measured at 595 nm using an ELISA reader. Untreated bacterial cultures served as the negative control.^50^

### Scanning Electron Microscopy (SEM)

To assess the effect of treatments on the cellular structure of CPR-*Kp*, SEM imaging was performed (27). CPR-*Kp* cells were exposed to OdEO-PMB, OvEO-PMB, or PMB at 2 μg/mL individually. A microbial growth control consisting of CPR-*Kp* cultured in liquid BHI medium was included. After treatment, the cells were fixed in a 2.5% glutaraldehyde solution and dehydrated stepwise using ethanol solutions of increasing concentrations (30%, 50%, 70%, and 100% v/v) for 10 min at each step. After dehydration, 20 μL of the sample was placed onto glass coverslips (0.8 × 0.8 cm). Once dried, the coverslips were coated with a thin layer of gold. A scanning electron microscope (JSM-6380LV, JEOL, USA) was used for imaging at the Multiuser Center for Analysis of Biomedical Phenomena (CMABio-UEA).^51^

### Hemolysis Assay

An ex vivo mouse blood cell assay was conducted to evaluate the toxic effects of OvEO-PMB, OdEO-PMB, and their individual components by assessing hemolytic activity.^34^ In this assay, 100 μL of freshly collected blood was combined with 100 μL of each treatment, either as single compounds or in combination, and incubated at room temperature for 4 h. After incubation, the samples were centrifuged at 2500 rpm for 5 min, and the supernatant was collected. The optical density of the supernatant was measured at 595 nm using an iMark Microplate Absorbance Reader. Triton X-100 (0.1%, v/v) was used as a positive control, while Dulbecco’s phosphate-buffered saline (D-PBS) served as a negative control. The hemolysis rate was determined using the following formula.

### In Vivo Toxicity Assay

The wild-type nematode *C. elegans* (N2) was used to assess the safety and toxicity of OdEO-PMB, OvEO-PMB, and PMB at 64 μg/mL. Initially, nematodes were cultured on nematode growth medium (NGM) and subjected to a bleaching protocol in which alkaline hypochlorite and sodium hydroxide were used to isolate embryos. These embryos were incubated at 16 °C on NGM plates until they reached the young adult (L4) stage. Next, groups of 20–30 nematodes were transferred to 24-well plates containing 2 mL of treatment solutions prepared in M9 liquid media. Tigecycline (TGC) at 64 μg/mL served as a reference treatment, while M9 medium alone was used as a negative control. Nematode viability was monitored every 24 h for 5 days at 16 °C, with mortality defined as the absence of movement in response to physical stimulation.^52^

### In vivo Infection Model

The *C. elegans* strain AU37 (glp-4; sek-1) was synchronized to ensure uniform developmental stages. Nematodes at the L4 larval stage were exposed to CPR-*Kp* (1.5 × 10^8^ CFU/mL) for 4 h to induce infection. After exposure, the nematodes were rinsed with M9 buffer to eliminate residual bacteria and then transferred to culture plates (20–30 nematodes per well). The treatments included OdEO-PMB, OvEO-PMB, and TGC, which served as reference antibiotics. Nematode survival was monitored every 24 h for 5 days at 16 °C, with mortality defined by a lack of response to physical stimulation. The experimental controls consisted of infected, untreated nematodes (positive control) and uninfected nematodes maintained in M9 buffer (negative control).^53^

### Statistical Analysis

To determine the differences among the experimental groups, one-way ANOVA and Tukey’s multiple comparison test were performed. The survival of *C. elegans* was analyzed using Kaplan–Meier survival curves, and statistical significance was assessed by conducting a log-rank test to compare survival distributions between groups. All statistical analyses and graphical representations were performed using the R programming language.^54^ For all tests, the results were considered to be statistically significant at *p* < 0.05.

## Supplementary Material



## Data Availability

Data supporting this study are available upon request from the corresponding author.All data are available from the corresponding authors upon reasonable request.

## Notes

Chromatographic profiles and mass spectra of the main constituents of *O. diospyrifolia* essential oil (OdEO) and *O. velloziana* essential oil (OvEO) (Figures S1 and S2) (PDF)
