# Supplementary material for: Overcoming Polymyxin Resistance in Klebsiella pneumoniae with Ocotea Essential Oils: Insights from In Vitro and In Vivo Analyses
Source: ACS Omega. 2025 Nov 26;10(48):58963–74. doi: 10.1021/acsomega.5c07706 (PMC12771118; doi:10.1021/acsomega.5c07706)
Supplement: Supplementary file 1 [file ao5c07706_si_001.pdf]

OVERCOMING POLYMYXIN RESISTANCE IN *Klebsiella pneumoniae* WITH OCOTEA ESSENTIAL OILS: INSIGHTS FROM IN VITRO AND IN VIVO ANALYSES

*Izadora D Faccin[a #], Julia P Arantes[a], Mariana C Sturaro[a], Eduardo J Coutinho[b], Danielle C da Cruz do Nascimento[b], Claudia A L Cardoso[b], Flavio M Alves[c], Shaline S L Fernandes[b], Nathalia da S Damacena[a], Gleyce H d A d Souza[a], Ruana C C da Silva[a], Luana Rossato[a], Euclésio Simionatto[b], Simone Simionatto[a *‡]*

[a] Laboratory of Research in Health Sciences, Federal University of Grande Dourados (UFGD), Rodovia Dourados–Itahum, Km 12, University City, ZIP Code 79804-970, Dourados, MS, Brazil

[b] Postgraduate Program in Natural Resources (PGRN), State University of Mato Grosso do Sul (UEMS), Dourados/Naviraí, Mato Grosso do Sul, 79950-000, Brazil.

[c] Laboratory of Botany, Institute of Biosciences (INBIO), Federal University of Mato Grosso do Sul (UFMS), Campo Grande, Mato Grosso do Sul 79070-900, Brazil

* simonesimionatto@ufgd.edu.br

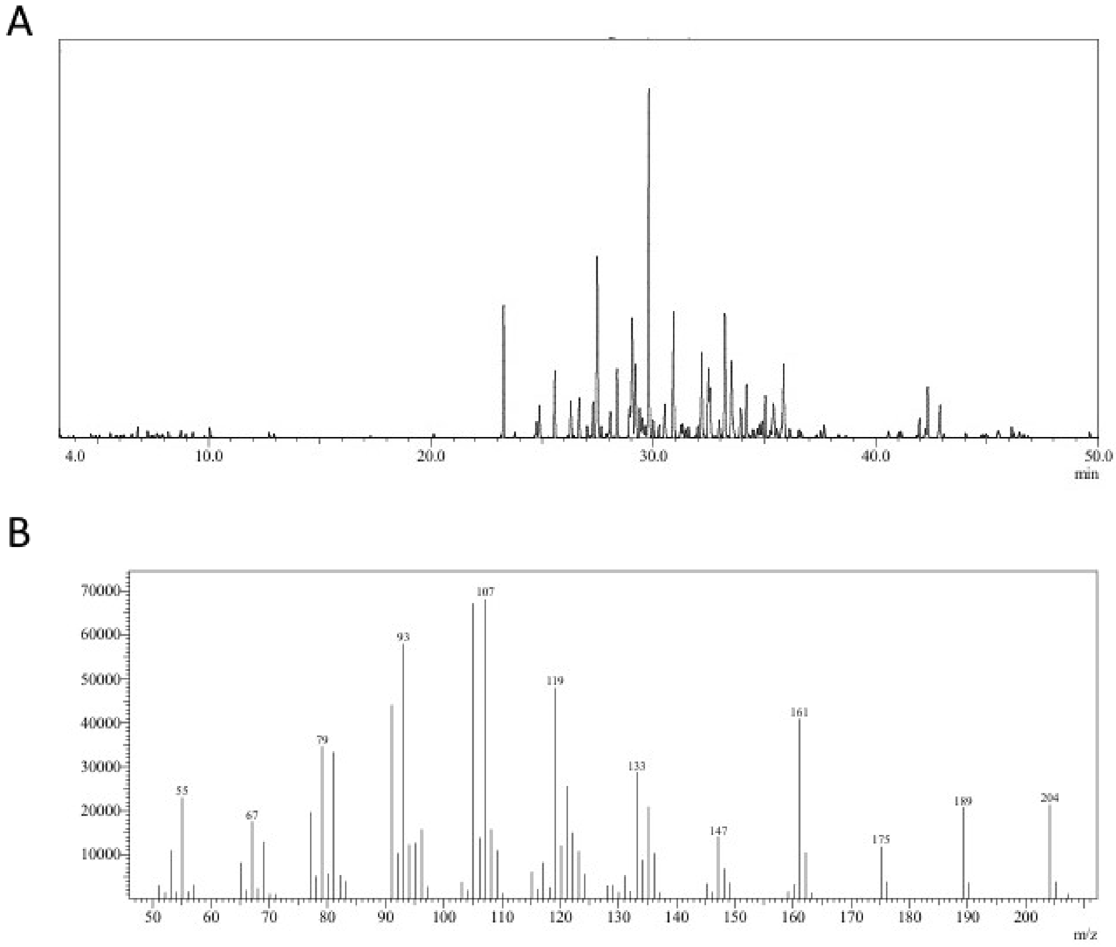

Figure S1. GC-MS of *Ocotea velloziana* essential oil (OvEO).

A) Chomatrogram of OvEO; B) Mass spectrum of the sesquiterpene viridiflorene of OvEO.

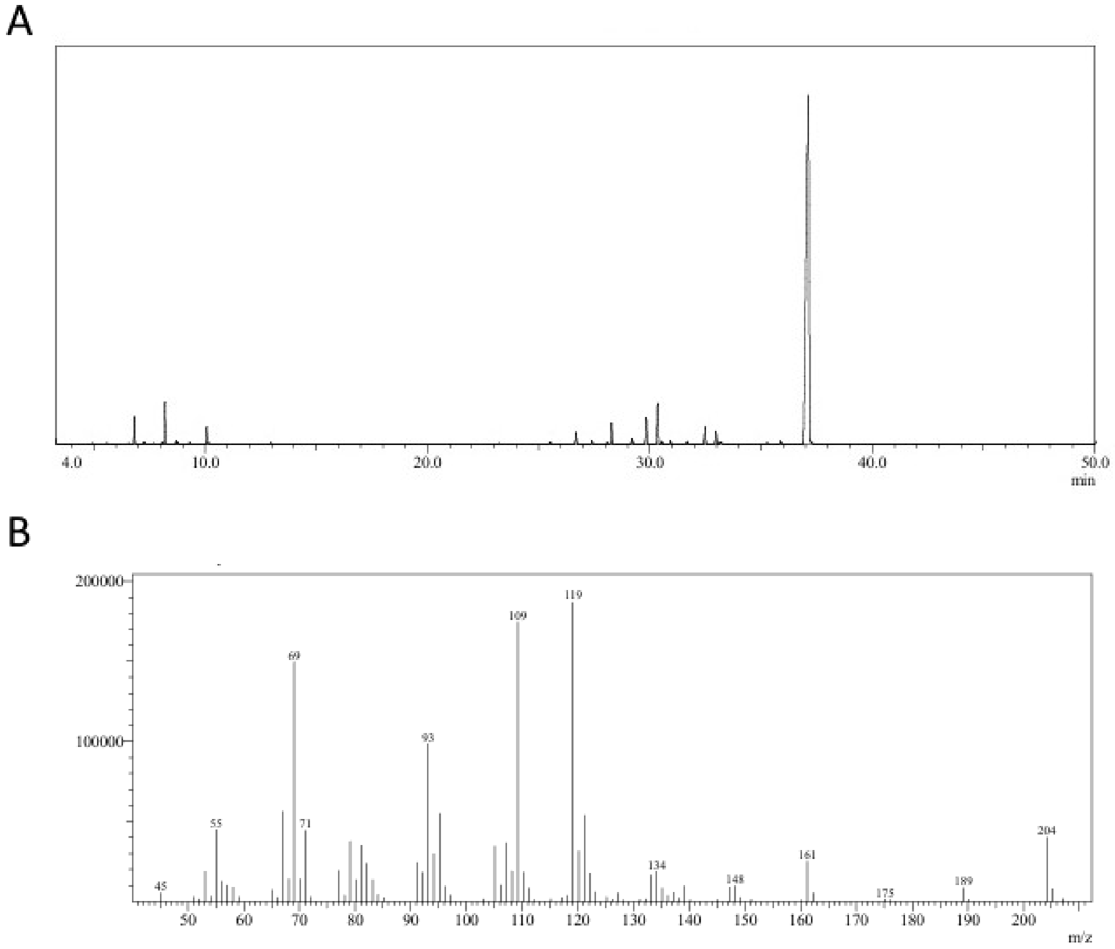

Figure S2. GC-MS of *Ocotea diospyrifolia* essential oil (OdEO).

A) Chomatrogram of OdEO; B) Mass spectrum of the sesquiterpene α-bisabolol of OdEO.
